# Left atrial fungal mass masquerading as myxoma: the importance of histopathological confirmation: a case report

**DOI:** 10.1186/s43044-025-00687-3

**Published:** 2025-09-18

**Authors:** MOHAMED MOUNIR NESNASSI, Asmae Benssied, Inasse Bargach, Siham Hallab, Safae Hilal, Ibtissam Fellat, Mohamed Cherti

**Affiliations:** Ibn Sina Hospital, Mohammed V University, Rabat, Morocco

**Keywords:** Infective endocarditis, Fungal cardiac mass, Fungal infective endocarditis, Left atrial myxoma

## Abstract

**Background:**

Infective endocarditis (IE) remains a serious and potentially life-threatening condition. Fungal infective endocarditis is a rare form of endocarditis that occur in immunodeficient patients. Atrial myxoma are a common cause of cardiac tumors in the left atrium. However, infective endocarditis is a differential diagnosis of cardiac masses in general and atrial myxoma particularly.

**Case summary:**

A 49-year-old male with subacute infective endocarditis presentation underwent an echocardiogram examination that showed a left atrial mass with features of atrial myxoma. However, the histological examination after surgical removal of the mass showed a fungal mass.

**Conclusion:**

This case shows that a fungal infective endocarditis may present with a cardiac mass sharing typical features with left atrial myxoma. It also shows the importance of histological examination for the final diagnosis and a prompt treatment.

## Introduction

Infective endocarditis (IE) remains a life-threatening disease despite advances in diagnostic and therapeutic strategies. According to the latest European Society of Cardiology (ESC) guidelines, when clinical presentation and blood cultures raise suspicion of infective endocarditis, echocardiography is usually the first imaging modality for the detection of vegetations, abscesses, and new valvular regurgitations being major criteria for diagnosis [1]. The histopathological examination is sometime required to further confirm the diagnosis. Although bacterial pathogens remain the most common etiologic agents, fungal IE accounts for less than 10% of cases and is associated with a particularly poor prognosis [1, 2]. Fungal endocarditis predominantly affects immunocompromised patients, those with prosthetic valves, or individuals with a history of intravenous drug use [2, 3]. For this reason, blood culture should look for both bacterial and fungal infections especially in some patients. The clinical and imaging presentation of fungal cardiac masses can mimic benign primary tumors such as atrial myxomas, complicating the diagnostic process [4, 5].

We report a rare case of left atrial fungal infective endocarditis in an immunocompetent patient, initially misdiagnosed as a myxoma, highlighting the importance of considering infectious etiologies in the differential diagnosis of intracardiac masses.

## Case presentation

We report the case of a 49-year-old male without medical history. He had a dental extraction performed 10 months prior. The patient presented with a two-month history of prolonged fever, headaches, night sweats, anorexia, weight loss, and fatigue. He also reported joint pain, mainly in the knees, and more recently palpitations and worsening dyspnea (NYHA class III).

Physical examination revealed a febrile state without cutaneous lesions. Cardiac auscultation detected a mitral regurgitation murmur. No signs of heart failure or embolic cutaneous manifestations were noted.

**Fig. 1:**
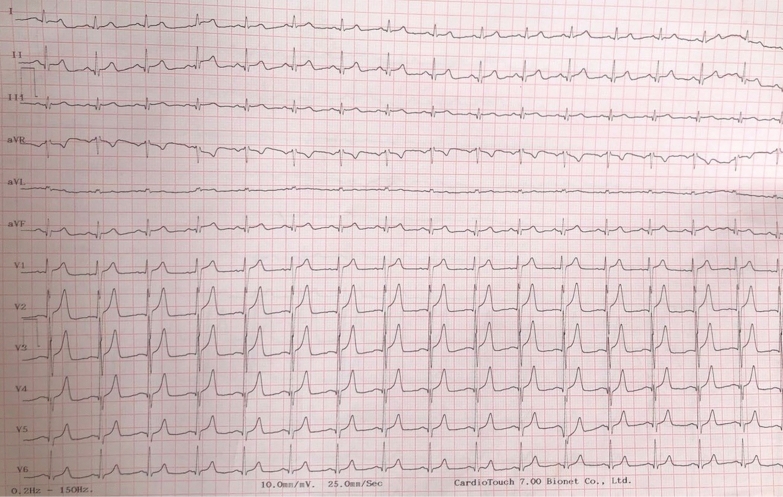
ECG of the patient

The electrocardiogram (ECG) showed a sinus rhythm at 85 beat per minute, a normal PR segment at 140 ms and normal QRS complexes without repolarization abnormalities (Fig. 1).

Laboratory tests showed anemia (hemoglobin at 9.9 g/dL), leukocytosis (16 420/mm^3^ decreasing to 13 500/mm^3^ with treatment), elevated CRP (111.9 mg/L, decreasing to 26 mg/L with treatment), procalcitonin at 0.26 µg/l, elevated ferritin levels at 1131 ng/ml, positive rheumatoid factor, 2 positive blood cultures after 1 day and 6 h for *Streptococcus Acidominumus* and another one positive blood culture *for Staphylococcus hominis* that was considered as contaminant.

**Fig. 2:**
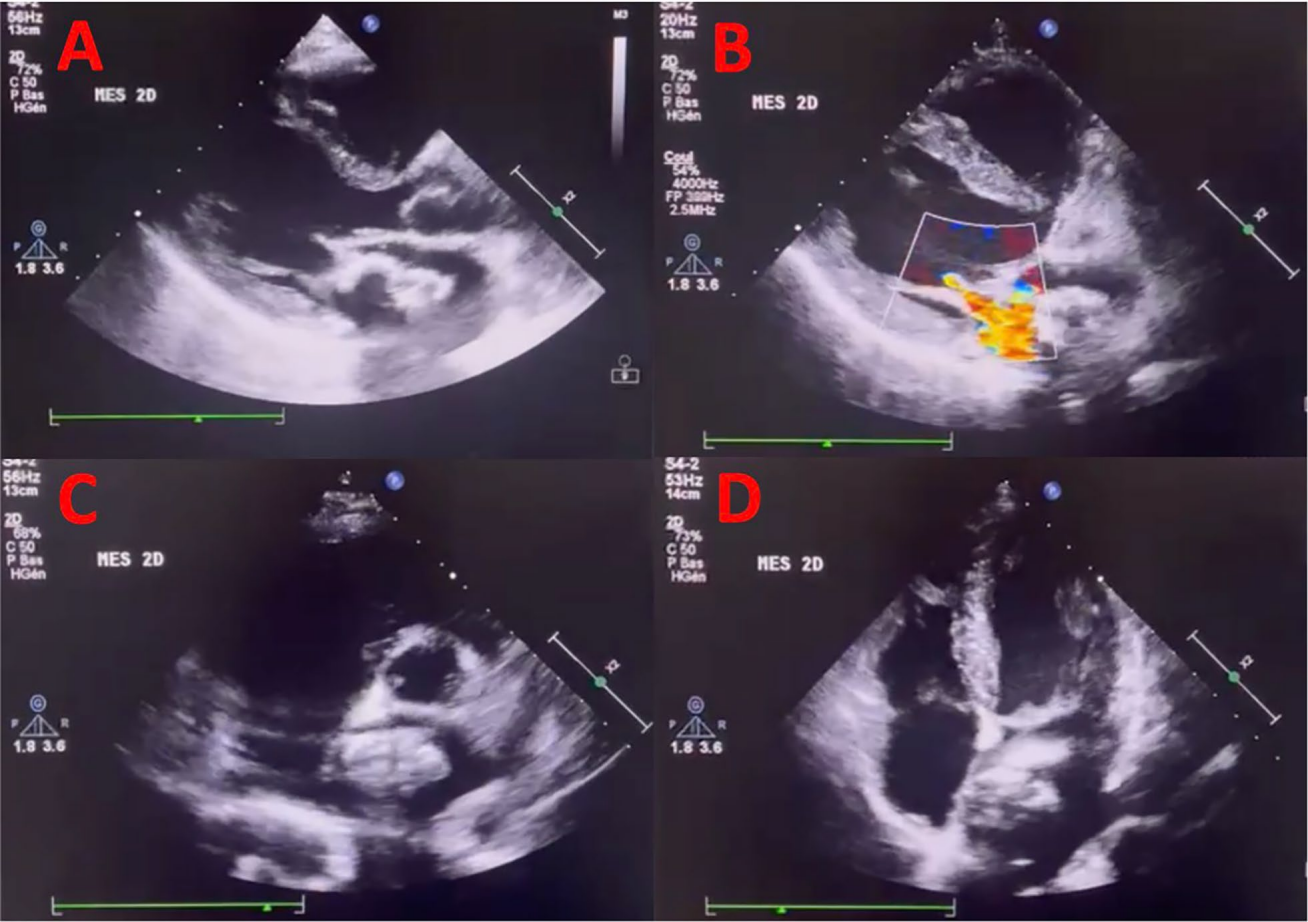
Transthoracic echocardiogram showing the left atrial mass. **A** Parasternal long axis showing the mass attached to the mitral valve. **B** Parasternal long axis showing mitral regurgitation. **C** Parasternal short axis showing the mass attached to the atrial septum. **D** Apical 4 chamber view showing the mass mimicking an atrial myxoma

Transthoracic Echocardiography (TTE) (Fig. 2) found a heterogeneous mobile left atrial mass attached to the interatrial septum and mitral valve with a diameter of 35 mm without mitral flow obstruction. The mean mitral gradient was at 2 mmHg. It also showed a central moderate mitral regurgitation (effective regurgitant orifice area (EROA) at 0.3 cm^2^ and a regurgitant volume at 48 ml), a left ventricular ejection fraction (LVEF) at 50% with end diastolic diameter at 54 mm. Left atrium was mildly dilated with a surface at 22 cm^2^. The rest of the echocardiogram was normal with no signs of pulmonary hypertension or pericardial effusion.

Based on the presence of positive blood cultures and the new onset of mitral regurgitation, the diagnosis of definite infective endocarditis was established according to the modified Duke/ESC criteria and the patient received intravenous antibiotics (Ceftriaxone 2 g/day for 15 days) adapted to the culture sensitivities with marked clinical improvement (the patient became afebrile after 2 days and the inflammatory markers decreased progressively with CRP levels reaching 26 mg/ml after 10 days).

**Fig. 3:**
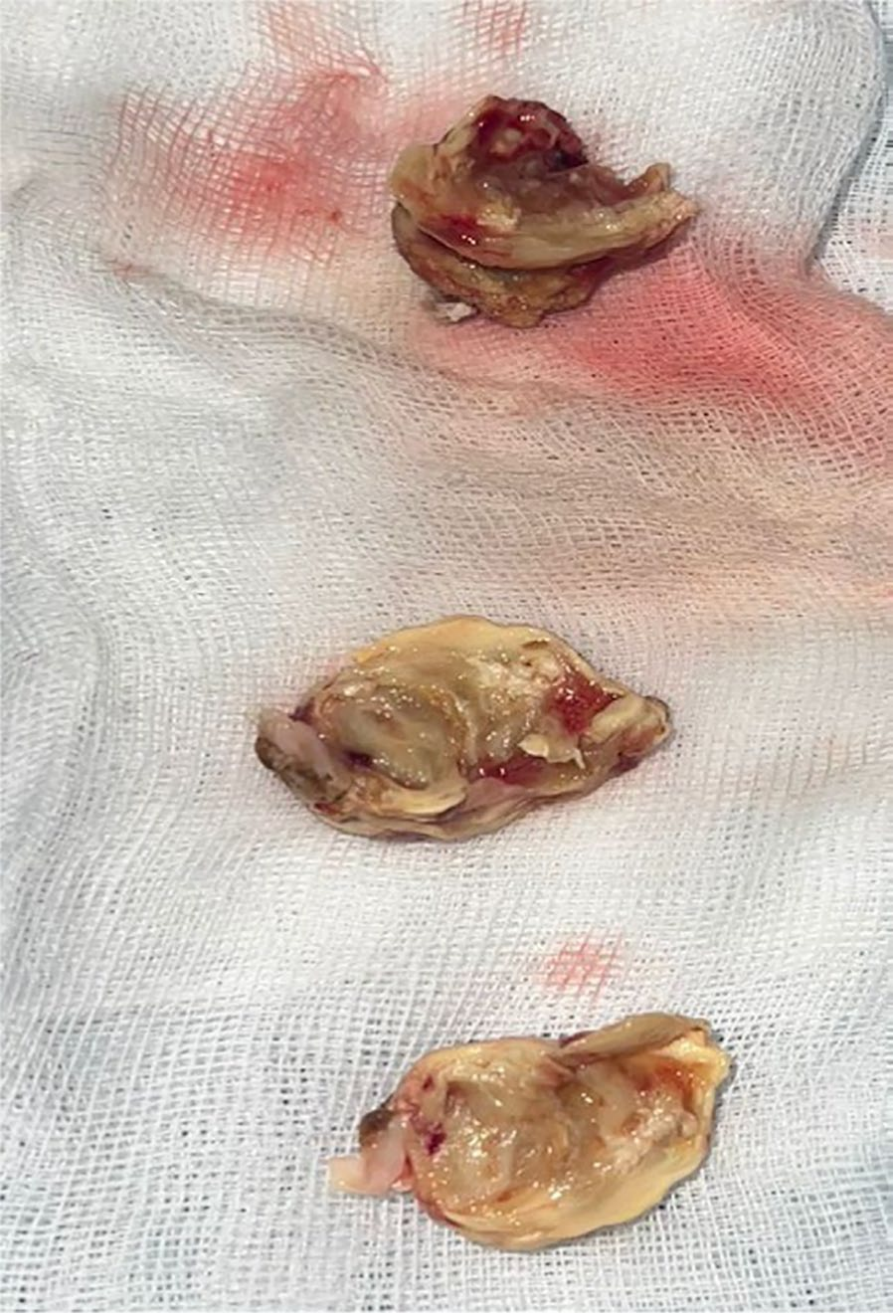
macroscopic aspect of the mass after its surgical removal

However, the cardiac mass was not a typical image of valvular vegetation. Because of its typical location, the diagnosis of left atrial myxoma was evoked and the patient underwent surgery after 16 days of hospitalization. Due to limited local availability, transesophageal echocardiography was not performed in order to avoid delaying surgery. The cardiac mass was successfully removed (Fig. 3) with a macroscopic aspect of a mass of 6 g and measuring 25 mm by 15 mm, calcified and grayish yellow in color. Because of the limited leaflet destruction, the mitral valve was not replaced but repaired with a patch and annuloplasty. The histological examination showed primarily fibrin-like material with fibrous organization and calcium rearrangement. Filamentous mycotic elements with spore-forming elements were also present.

The histopathological examination proved the diagnosis of fungal endocarditis according to the modified Duke/ESC criteria. The patient received antifungal treatment with lipid-associated amphotericin B 300 mg (5 mg/kg) daily for two weeks along with Ceftriaxone then the patient was discharged from hospital after a total hospital stay of one month. While on antifungal therapy, we closely monitored kidney function tests and serum electrolytes. Blood cultures looking for both bacterial and fungal agents were repeated at the end of the treatment and were negative. At the one-month follow-up after surgery, echocardiographic evaluation demonstrated a marked reduction in mitral regurgitation, with only minimal residual flow observed.

Given that systemic antifungal therapy was discontinued postoperatively, and considering the risk of relapse in fungal infective endocarditis, we planned a close clinical and laboratory follow-up, including periodic monitoring of inflammatory markers and careful assessment for any signs of recurrence. An echocardiographic evaluation is scheduled at three months to ensure proper cardiac function and early detection of potential vegetations or complications. This follow-up strategy allows timely intervention if any relapse occurs.

## Discussion

Primary cardiac tumors are rare, and myxomas represent the most common type, accounting for nearly half of them. Myxomas predominantly arise from the interatrial septum of the left atrium and typically present with obstructive cardiac symptoms, embolic events, or systemic constitutional symptoms such as fever and weight loss [6]. Conversely, infective masses of the left atrium, particularly fungal infections, are extremely rare and often under-recognized. Fungal masses in the left atrium may clinically and radiologically can mimic myxomas, posing a significant diagnostic challenge.

Although myxomas are relatively frequent among cardiac masses, left atrial fungal infections are exceptionally rare, particularly in immunocompetent individuals without prosthetic valves or a history of intravenous drug use. Fungal endocarditis represents less than 10% of all infective endocarditis cases, with *Candida albicans* being the predominant pathogen [2, 3, 7].

The clinical spectrum of fungal endocarditis is evolving due to the increasing use of immunosuppressants, cardiac devices, transthoracic echocardiography and the introduction of newer antifungal agents. Colonization (skin, gastrointestinal) and subsequent invasion are necessary steps in FE. Afterwards, the release of various hydrolytic enzymes, hemolysin, proteases, lipases and candida surface protein mediate the adhesion to the valve [8].

When infections do involve the atria, they are more frequently bacterial and localized to the endocardium or valves. Left atrial fungal masses have only been reported sporadically in the literature [7, 9].

The diagnostic confusion arises when a myxoma displays atypical echocardiographic features. Classic myxomas are pedunculated, mobile, and attached to the fossa ovalis region of the interatrial septum. However, atypical myxomas may appear sessile, irregular, and broad-based, sometimes lacking the typical "ball-valve" motion and resulting in a more infiltrative appearance on echocardiography [10]. These atypical characteristics overlap with those of thrombi and infective vegetations.

Fungal infections of the left atrium typically appear on echocardiography as heterogeneous, poorly defined masses, sometimes associated with mural thrombus formation or adjacent tissue destruction [11]. Transesophageal echocardiography (TEE) is more sensitive than transthoracic echocardiography (TTE) for detecting vegetations and fungal masses [12]. Nevertheless, the absence of a stalk, the irregular shape, and rapid evolution of the mass may orient toward an infectious origin rather than a tumoral one [10, 12].

On macroscopic examination, myxomas are generally gelatinous, polypoid, and well circumscribed, sometimes displaying areas of hemorrhage [6]. In contrast, fungal masses are often friable, large, and poorly circumscribed [9].

Histologically, myxomas are composed of scattered polygonal or stellate cells embedded in a myxoid stroma, without any evidence of organisms [6]. In contrast, fungal masses show thrombotic material with infiltration of yeast and hyphal forms, which can be demonstrated by Periodic Acid-Schiff (PAS) staining or Grocott methenamine silver stain [9].

Thus, histopathological analysis remains the gold standard for definitive diagnosis in such cases [9]. Despite appropriate antifungal treatment with amphotericin B, the prognosis of fungal endocarditis remains poor, often complicated by embolic events and sepsis [2, 9].

In our case, the echocardiographic features strongly suggested the presence of a cardiac myxoma, given its relative frequency. However, the final diagnosis revealed a fungal mass, a much rarer and less well-known entity. This rarity can lead to delays in diagnosis and treatment, especially since fungal infective endocarditis requires more urgent surgical intervention than a myxoma due to its generally poorer prognosis if not managed promptly. In our case, fungal endocarditis was not initially suspected, particularly given that the patient was immunocompetent and had no apparent risk factors for developing such an infection. This raises the question: Did the initial bacterial infection of the mitral valve create a predisposition for secondary fungal colonization, ultimately leading to the development of the mass? Fortunately, surgery was performed early in our patient, resulting in a favorable outcome.

Current guidelines generally recommend combined surgical intervention plus prolonged systemic antifungal therapy (often ≥ 6 weeks), with step-down to azole therapy when appropriate [4, 9]. However, these recommendations are largely based on cohorts including immunocompromised patients or those with prosthetic valves. In our case, the patient was immunocompetent, and we hypothesized that the fungal infection was facilitated by a preceding bacterial endocarditis. Notably, blood cultures remained negative after surgery, and the patient showed rapid clinical improvement with antibiotic therapy alone prior to any antifungal treatment, suggesting a low likelihood of fungal recurrence. Published reports also emphasize that the optimal duration of intravenous antifungal therapy should be individualized and may not always exceed four weeks in selected cases, highlighting the need for case-by-case assessment [4, 9]. Additionally, considerations such as drug toxicity, cost, and monitoring requirements—including renal function and electrolytes—further support tailored therapy. Thus, in this particular scenario, we opted for a limited antifungal approach while maintaining close clinical and laboratory follow-up, considering the relatively low risk of recurrence**.**

The key takeaway from this case is that a fungal mass should be considered as a differential diagnosis of atrial myxoma, particularly in the presence of other signs suggestive of infective endocarditis.

## Conclusions

This case highlights the diagnostic challenge of differentiating between infective endocarditis and primary cardiac tumors when encountering atypical intracardiac masses. Left atrial fungal endocarditis remains exceedingly rare, especially in immunocompetent patients without prosthetic valves or intravenous drug use. The echocardiographic appearance mimicking an atrial myxoma led to surgical resection, which provided the definitive diagnosis through histopathological analysis. According to ESC guidelines, the management of fungal infective endocarditis requires a combination of antifungal therapy and surgical intervention to improve outcomes. Clinicians should maintain a high index of suspicion for infectious etiologies in cases of atypical intracardiac masses to guide timely and appropriate management.

## Data Availability

No datasets were generated or analysed during the current study.

## Abbreviations

IE: Infective endocarditis
ESC: European Society of Cardiology
NYHA: New York Heart Association
TTE: Transthoracic Echocardiography
LVEF: Left ventricular ejection fraction
TEE: Transesophageal echocardiography
PAS: Periodic Acid-Schiff
